# Relationship between Adipokines and Cardiovascular Ultrasound Parameters in Metabolic-Dysfunction-Associated Fatty Liver Disease

**DOI:** 10.3390/jcm10215194

**Published:** 2021-11-07

**Authors:** Abdulrahman Ismaiel, Mihail Spinu, Livia Budisan, Daniel-Corneliu Leucuta, Stefan-Lucian Popa, Bogdan Augustin Chis, Ioana Berindan-Neagoe, Dan Mircea Olinic, Dan L. Dumitrascu

**Affiliations:** 1 2nd Department of Internal Medicine, “Iuliu Hatieganu” University of Medicine and Pharmacy, 400006 Cluj-Napoca, Romania; abdulrahman.ismaiel@yahoo.com (A.I.); bogdan_a_chis@yahoo.com (B.A.C.); ddumitrascu@umfcluj.ro (D.L.D.); 2 Medical Clinic No. 1, “Iuliu Hatieganu” University of Medicine and Pharmacy, 400006 Cluj-Napoca, Romania; spinu_mihai@yahoo.com (M.S.); danolinic@gmail.com (D.M.O.); 3 Research Center for Functional Genomics, Biomedicine and Translational Medicine, “Iuliu Hațieganu” University of Medicine and Pharmacy, 400337 Cluj-Napoca, Romania; lbudisan@yahoo.com (L.B.); ioana.neagoe@umfcluj.ro (I.B.-N.); 4 Department of Medical Informatics and Biostatistics, “Iuliu Hatieganu” University of Medicine and Pharmacy, 400349 Cluj-Napoca, Romania; dleucuta@umfcluj.ro; 5 Research Center for Advanced Medicine-Medfuture, Iuliu Hatieganu University of Medicine and Pharmacy, 23 Marinescu Street, 400337 Cluj-Napoca, Romania; 6 Department of Functional Genomics and Experimental Pathology, The Oncology Institute “Prof. Dr. Ion Chiricuta”, 400015 Cluj-Napoca, Romania; 7 Interventional Cardiology Department, Emergency Clinical Hospital, 400006 Cluj-Napoca, Romania

**Keywords:** metabolic-dysfunction-associated fatty liver disease (MAFLD), hepatic steatosis, SteatoTest, adipokines, adiponectin, visfatin, cardiovascular disease

## Abstract

(1) Background: The role of adipokines such as adiponectin and visfatin in metabolic-dysfunction-associated fatty liver disease (MAFLD) and cardiovascular disease remains unclear. Therefore, we aim to assess serum adiponectin and visfatin levels in MAFLD patients and associated cardiovascular parameters. (2) Methods: A cross-sectional study involving 80 participants (40 MAFLD patients, 40 controls), recruited between January and September 2020, was conducted, using both hepatic ultrasonography and SteatoTestTM to evaluate hepatic steatosis. Echocardiographic and Doppler parameters were assessed. Serum adipokines were measured using ELISA kits. (3) Results: Adiponectin and visfatin levels were not significantly different in MAFLD vs. controls. Visfatin was associated with mean carotid intima-media thickness (*p*-value = 0.047), while adiponectin was associated with left ventricular ejection fraction (LVEF) (*p*-value = 0.039) and E/A ratio (*p*-value = 0.002) in controls. The association between adiponectin and E/A ratio was significant in the univariate analysis at 95% CI (0.0049–0.1331, *p*-value = 0.035), but lost significance after the multivariate analysis. Although LVEF was not associated with adiponectin in the univariate analysis, significant values were observed after the multivariate analysis (95% CI (−1.83–−0.22, *p*-value = 0.015)). (4) Conclusions: No significant difference in serum adiponectin and visfatin levels in MAFLD patients vs. controls was found. Interestingly, although adiponectin levels were not associated with LVEF in the univariate analysis, a significant inversely proportional association was observed after the multivariate analysis.

## 1. Introduction

Although fatty liver disease is mainly associated with structural and functional liver alterations, as well as increased liver-related morbidity and mortality as a result of possible progression to cirrhosis, liver failure and, ultimately, hepatocellular carcinoma, it is also well known to exert several extrahepatic manifestations [1,2,3,4]. Lately, a significant increase in the worldwide prevalence of metabolic-dysfunction-associated diseases, including fatty liver disease, type 2 diabetes mellitus, dyslipidemia and obesity, has been documented [5]. Despite the importance of treating fatty liver disease, we still remain without approved pharmacotherapies [6].

Recently, the term metabolic-dysfunction-associated fatty liver disease (MAFLD) was suggested to replace the previously known non-alcoholic fatty liver disease (NAFLD) which is defined by the presence of steatosis in >5% of hepatocytes, associated with insulin resistance (IR) [1,2,7]. On the other hand, MAFLD is defined by the presence of fatty liver, in addition to one of the following three criteria: overweight/obesity, type 2 diabetes mellitus, or confirmed metabolic risk dysregulation [8,9]. Therefore, the terms NAFLD and MAFLD should not be used interchangeably due to the diagnostic criteria differences between the terms. Multiple studies reported fatty liver disease as an independent risk factor associated with increased cardiovascular disease (CVD)-related morbidity and all-cause mortality [10,11]. This association can be attributed to several possible pathogenic factors increasing the CV risk in MAFLD, including IR, systemic inflammation, cytokines, oxidative stress, adipokines, hepatokines, genes and intestinal microbiota [12]. Nevertheless, other studies demonstrated that fatty liver disease per se is not causally related to an increased cardiovascular (CV) risk, implying an essential role of plasma lipids in this relationship.

Interestingly, a recently published study demonstrated that MAFLD was found to be associated with a higher risk for cardiovascular mortality and risk of all-cause mortality [13,14]. However, NAFLD per se did not increase the risk of all-cause deaths after adjusting for metabolic risk factors. Therefore, possible pathogenic factors linking cardiovascular disease to fatty liver disease should be reassessed using the newly defined MAFLD criteria due to possible result differences.

Adipose tissue, known to act as a highly active endocrine tissue, produces peptides known as adipokines with autocrine, paracrine and endocrine functions. Lately, an increased interest in evaluating adipokines such as adiponectin and visfatin in several obesity-related diseases, including fatty liver disease and CVD, has been demonstrated [5,15]. Adiponectin is the most abundant peptide secreted by adipocytes. It was also found to be secreted from other cells, including skeletal and cardiac myocytes, in addition to endothelial cells. Reduction in adiponectin levels has a crucial role in obesity-related pathologies, such as insulin resistance, type 2 diabetes mellitus and CVD [16]. Although serum adiponectin levels were demonstrated to be similar in non-alcoholic fatty liver (NAFL) patients and controls based on liver histology, hypoadiponectinemia may exert an essential role in the progression from NAFL to non-alcoholic steatohepatitis (NASH) [17].

Visfatin is also secreted from adipocytes, as well as lymphocytes, neutrophils, monocytes, hepatocytes and pneumocytes [18]. Multiple pathways affected by visfatin include oxidative stress response, insulin resistance and inflammation [19,20,21]. A recently published meta-analysis evaluated serum visfatin levels in NAFLD, demonstrating no significant association [22]. Furthermore, increased visfatin levels were found to be associated with atherosclerotic disease and coronary artery disease (CAD), pathologies demonstrated to be among the main mortality causes in fatty liver disease [23,24,25,26].

Currently, the literature lacks studies evaluating serum adiponectin and visfatin levels in MAFLD patients using the newly defined criteria. Moreover, adiponectin and visfatin were both found to be associated with CVD, being responsible for most deaths among fatty liver disease patients, mainly due to ischemic heart disease [27,28]. Therefore, we conducted an observational cross-sectional study aiming to evaluate serum adiponectin and visfatin levels in MAFLD patients vs. controls, as well as their possible associations with cardiovascular parameters assessed by echocardiography and Doppler ultrasound. We hypothesized that adiponectin and visfatin can predict cardiovascular risk, mainly systolic and diastolic dysfunctions.

## 2. Materials and Methods

### 2.1. Study Participants and Setting

This is an observational cross-sectional study. Subjects were enrolled between January 2020 and September 2020 using non-probability consecutive sampling of eligible subjects. Inclusion criteria included subjects ≥18 and <65 years old. Patients admitted to the Clinical Emergency County Hospital of Cluj-Napoca, Romania, fulfilling the diagnostic criteria of MAFLD were included in the MAFLD group [9]. Hepatic steatosis was evaluated using both hepatic ultrasonography and SteatoTestTM (BioPredictive) simultaneously for the entire sample population (MAFLD patients and controls) at inclusion. Subjects had to present hepatic steatosis using both ultrasonography and SteatoTestTM (BioPredictive) in order to be included in the MAFLD group. Otherwise, they were excluded from the MAFLD group. Control participants were primarily healthy hospital staff not fulfilling the diagnostic criteria for MAFLD.

Exclusion criteria for both control and MAFLD groups included subjects with the presence of other secondary causes of hepatic steatosis evaluated by assessing alcohol consumption through AUDIT and CAGE questionnaires, use of hepatotoxic medications such as glucocorticoids, isoniazid, methotrexate, amiodarone and tamoxifen within 1 year from being enrolled in the study, positive hepatitis B or C virus serology, elevated ferritin concentration ≥1000 μg/L, significantly positive immunology titers for anti-smooth muscle antibody or antimitochondrial antibody, or a previous diagnosis of persistent secondary cause known for chronic liver disease. Moreover, individuals with benign or malignant liver tumor, coexistent liver disease, acute hemolytic diseases, acute inflammatory pathologies such as deep venous thrombosis, Ulcerative Colitis or Crohn’s Disease, systemic lupus erythematosus, active malignancies, acute infections (dental, urinary, pulmonary, flu, COVID-19, etc.), active pulmonary exacerbations such as COPD exacerbation or asthma, failing to fast for a minimum of 12 h before blood sampling and refusing participation were excluded. The local ethical and research committee of “Iuliu Hatieganu” University of Medicine and Pharmacy Cluj-Napoca approved the performance of this study (no. 486/21 November 2019), which was conducted according to the 1975 Helsinki Declaration guidelines and revised in 2013. All included participants completed a written informed consent.

### 2.2. General Definitions

The diagnosis of MAFLD was based on the newly defined criteria [9]. The definition of hypertension was considered according to the 2020 International Society of Hypertension Global Hypertension Practice Guidelines [29]. The diagnosis of diabetes and prediabetes were determined according to the American Diabetes Association recommendations—Classification and Diagnosis of Diabetes: Standards of Medical Care in Diabetes (2021) [30]. Dyslipidemia was identified according to the National Cholesterol Education Program guidelines [31].

### 2.3. Hepatic Ultrasonography

Ultrasonographic evaluation of hepatic steatosis was performed using GE LOGIQ S7 Expert by an experienced physician who was blinded to the aims of the study, patients’ diagnosis and labs. Subjects were requested to fast for a minimum of 8 h before performing the ultrasound evaluation, where a subcostal and intercostal approach was used to assess the liver parenchyma. Participants were evaluated in a supine position and in a modified, slightly oblique position with their right arm placed above their head and their right leg stretched. The following criteria were used in order to evaluate for hepatic steatosis: (1) ultrasonographic contrast between liver and right kidney parenchyma; (2) hepatic brightness; (3) ultrasound deep attenuation penetration into the deep portion of the liver and impaired diaphragmatic visualization; and (4) lumen narrowing and impaired intrahepatic vessels borders’ visualization [32].

### 2.4. Echocardiography

A comprehensive echocardiographic assessment was conducted by a board-certified cardiologist who was blinded to the study aims, patients’ diagnosis and labs, independent of the adipokines evaluation, using a GE Vivid q Ultrasound Machine, 11.2.0 b.40 software. The current recommendations and guidelines were used for measuring and interpreting our assessed parameters, including M-mode, 2-dimensional, conventional color and Doppler ultrasonography [33,34,35,36,37,38]. We used a dedicated software for automated calculation of end-systolic volume (ESV) and end-diastolic volume (EDV) from the 2- and 4-chamber apical views, as well as left ventricular ejection fraction (LVEF), while verification and correction of precision for the detected borders were conducted. Using apical 4-chamber views, we obtained Doppler-derived transmitral inflow profiles with a sample volume of 2 mm placed between the mitral leaflet tips. The early (E) and late (A) peak velocity phases of the mitral inflow were measured from the mitral inflow Doppler evaluation, while an automatic calculation was used for the E/A ratio. Moreover, also using the 4-chamber view, we calculated the LV myocardial velocities through Tissue Doppler imaging (TDI) with a sample volume being placed at the septal mitral annulus. We also calculated the early diastolic (e′) and late diastolic peak velocity phases using the pulsed-wave TDI, while the E/e′ ratio was automatically calculated.

### 2.5. Laboratory Analysis

We obtained blood samples through venipuncture and collected them in vacutainer tubes following 12 h of overnight fasting. We followed the recommended protocols for blood sampling and for analyzing blood samples.

#### 2.5.1. Adipokines

For the adipokines’ analysis (adiponectin and visfatin), peripheral blood was collected on a clot activator. Blood was transported to the Research Center for Functional Genomic, Biomedicine and Translational Medicine, “Iuliu Hatieganu” University of Medicine and Pharmacy, Cluj-Napoca, Romania within 30 min from sampling for centrifugation for 15 min at 1000× *g* at 2~8 °C. The supernatant was collected to carry out the assay. Centrifuged blood samples were stored at −80 °C. The serum adiponectin analysis was performed using the BioVendor Adiponectin Human ELISA (Competitive) RD195023100 kit, while the serum visfatin analysis was performed using the Elabscience Human VF (Visfatin) ELISA Kit E-EL-H1763. All analyses were conducted as per the manufacturer’s instructions.

#### 2.5.2. FibroMax

Sera were separated and stored at 2 °C–8 °C for 1 day at most. Afterward, they were assayed for the ten serum biomarkers included in the FibroMax score. Adjustments for age, gender, weight and height of the achieved results were performed for obtaining the final score.

The serum levels of α2-macroglobulin, haptoglobin, apolipoprotein A1 were evaluated using nephelometry (BN ProSpec System from Siemens), while total bilirubin, gamma-glutamyltransferase (GGT), aspartate aminotransferase (AST), alanine aminotransferase (ALT), total cholesterol and triglycerides were assessed using spectrophotometry (Atellica from Siemens). Moreover, NaF/K2 oxalate spectrophotometry was used to assess plasma fasting glucose levels. Obtained values of the evaluated blood variables were completed into the BioPredictive network, where computed algorithms were performed.

### 2.6. Statistical Analysis

We used the R software environment for statistical computing and the graphics version 4.0.2 (R Foundation for Statistical Computing, Vienna, Austria) for carrying out the statistical analyses. Frequencies and percentages were used to report categorical data. For continuous data, normally distributed data were reported as mean (standard deviation, SD), while non-normally distributed data were reported as median (interquartile range, IQR). We used quantile–quantile plots to assess the normality of the distribution of the data. We used the *t*-test for independent samples of normally distributed data for comparing the clinical characteristics of the study population as per the categorized groups. Furthermore, the Wilcoxon rank-sum test was used for non-normally distributed data, while the chi-square test and Fisher’s exact test were used for categorical data. We used Fisher’s exact test in case expected frequencies were low, otherwise, we used chi-square tests. In order to evaluate the relationship between adiponectin and visfatin with several cardiovascular parameters, we performed Spearman’s correlations, followed by univariate and multivariate linear regression models to control for confounding factors including MAFLD vs. controls, gender, diabetes, mean systolic blood pressure (SBP) (mmHg), mean diastolic blood pressure (DBP) (mmHg), low-density lipoprotein (LDL) and triglycerides. For all conducted linear models, we checked the assumptions of residual normality using a quantile–quantile plot, heteroskedasticity using standardized residual vs. fitted values, the presence of high leverage, high residuals, or high influential points using standardized residuals vs. hat-values vs. Cook’s distance plot and the linearity relation of continuous variables with the outcome using component + residual plot. Moreover, we evaluated the presence of multicollinearity in multivariate models using correlation coefficients and variance inflation factors. We reported the regression results as model coefficients, 95% confidence interval (CI—computed with robust variance sandwich estimators in case of heteroskedasticity) and *p*-value. Furthermore, we performed multiple quantile regressions to better keep into account possible deviations from multiple linear model assumptions. Two-sided statistical tests were performed for all analyses. A *p*-value < 0.05 was considered to be statistically significant.

## 3. Results

### 3.1. General Characteristics and Laboratory Results

**Figure 1:**
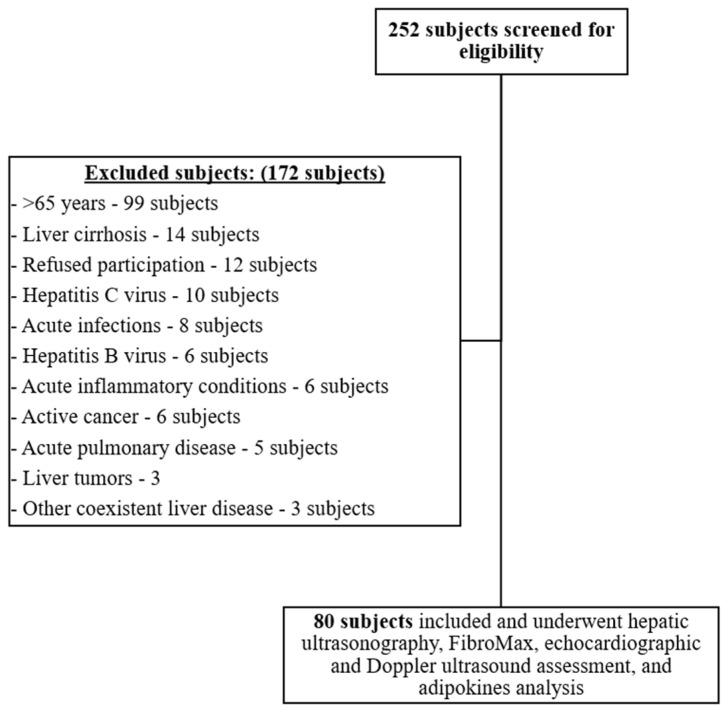
Flow diagram outlining the enrollment process of included and excluded participants.

The subjects screened for eligibility were 252, out of which 172 were excluded with reasons as demonstrated in Figure 1. A total of 80 Caucasian participants were included in our study’s final analysis. The participants’ general characteristics are outlined in Table 1.

Participants were divided into 2 groups, MAFLD patients and controls, equally, with 40 subjects in each group with a total mean age of 46 (ranging from 30 to 57). Gender distribution was 44 females (55%) and 36 males (45%), with no statistically significant difference (*p*-value = 1). MAFLD patients had a higher BMI, larger waist circumference, presence of diabetes and hypertension compared to controls (*p*-value < 0.001). No significant difference was found regarding smoking history among both groups (*p*-value = 0.963). Blood pressure measurements, including SBP, DBP, mean arterial pressure and pulse pressure, were all significantly higher in MAFLD patients compared to controls, with a *p*-value of <0.001, <0.001, <0.001 and 0.023, respectively. High-density lipoprotein (HDL) levels were significantly lower in MAFLD patients with a *p*-value of <0.001, with no significant difference regarding LDL (*p*-value = 0.083) or total cholesterol (*p*-value = 0.441) levels.

### 3.2. Hepatic Steatosis and Fibrosis Evaluation

A summary of the obtained hepatic steatosis, liver fibrosis and FibroMax scores is outlined in Table S1. Although all MAFLD patients presented with hepatic steatosis on ultrasonography and SteatoTest, only one participant from the control group had hepatic steatosis but did not fulfill the rest of the criteria for MAFLD diagnosis. A significant difference was found in all evaluated hepatic steatosis (FLI and HSI), liver fibrosis (APRI, FIB-4, BARD and NAFLD fibrosis score) and FibroMax scores between MAFLD patients and controls.

### 3.3. Adipokine Levels

No significant difference was found between the levels of the two evaluated adipokines in MAFLD patients and controls, adiponectin and visfatin, with a *p*-value of 0.097 and 0.26, respectively.

### 3.4. Cardiovascular Assessment

Multiple echocardiographic and Doppler ultrasound parameters were evaluated as summarized in Table 2. Structural parameters included higher mean carotid intima medica thickness (CIMT) (*p*-value < 0.001), left atrial diameter (*p*-value < 0.001), left ventricular diameter (*p*-value = 0.002), right ventricular diameter (*p*-value = 0.003), left ventricular posterior wall thickness (LVPWT) (*p*-value < 0.001), interventricular septal wall thickness (*p*-value < 0.001) and interatrial septal wall thickness (*p*-value = 0.018) in MAFLD patients vs. controls. Functional parameters included higher left ventricular end systolic volume (LVESV) (*p*-value < 0.001), left ventricular end diastolic volume (LVEDV) (*p*-value < 0.001), stroke volume (*p*-value 0.027), cardiac output (*p*-value = 0.029), late diastolic peak velocity (A) (*p*-value < 0.001), E/A ratio (*p*-value < 0.001) and E/e′ ratio (*p*-value = 0.004), as well as lower LVEF (*p*-value = 0.011), early diastolic peak velocity (E) (*p*-value < 0.001), early diastolic velocity (e′) (*p*-value < 0.001), e′/a′ (*p*-value < 0.001) in MAFLD patients vs. controls. No significant difference was observed in a′ vales (*p*-value = 0.265).

### 3.5. Adipokines and Cardiovascular Assessment

As demonstrated in Table S2, in the control group, visfatin levels were found to be inversely proportional and significantly associated with mean CIMT (*p*-value = 0.047), LVPWT (*p*-value = 0.003) and interventricular septal wall thickness (*p*-value = 0.008). Furthermore, in MAFLD patients, adiponectin was directly proportional and significantly related to A (*p*-value = 0.032), while, in controls, it was inversely proportional with LVEF (*p*-value = 0.039) and directly proportional with E (*p*-value = 0.003) and E/A ratio (*p*-value = 0.002). In all subjects, adiponectin levels were found to be significantly associated and inversely proportional with right ventricular diameter (*p*-value = 0.029) and LVPWT (*p*-value = 0.033) and directly proportional with E (*p*-value = 0.002).

We proceeded by assessing whether adiponectin and visfatin can be considered as potential biomarkers in evaluating E/A ratio, LVPWT, LVEF, CIMT and interventricular septal wall thickness by conducting several univariate and multivariate linear regression models adjusted for MAFLD, gender, diabetes, mean SBP (mmHg), mean DBP (mmHg), LDL and triglycerides, as reported in Table 3. No significant findings were demonstrated between adiponectin and LVPWT, nor between visfatin and LVPWT, CIMT and interventricular septal wall thickness in univariate and multivariate regression models. The association between adiponectin and E/A ratio was initially significant in the univariate linear regression analysis at 95% CI (0.0049–0.1331, *p*-value = 0.035). However, the significance was attenuated to non-significant levels after performing multivariate linear regression models. Interestingly, although the association between adiponectin and LVEF was not significant in the univariate analysis, significant values were reported after performing multivariate linear regression models with B-adjusted sandwich estimator of 95% CI (−1.83–−0.22, *p*-value = 0.015) and with B-adjusted quantile regression estimator of 95% CI (−1.97–−0.60, *p*-value = 0.011).

## 4. Discussion

Several articles, including systematic reviews and meta-analyses evaluated adiponectin and visfatin levels in NAFLD [17,22]. However, so far, to the best of our knowledge, no studies have evaluated these adipokines in patients with MAFLD using the newly defined diagnosis criteria. Moreover, although decreased adiponectin levels and increased visfatin levels are known to be associated with several cardiovascular diseases, these parameters were not assessed in MAFLD patients for their possible use as potential biomarkers. Therefore, in this observational study, we aim to assess adiponectin and visfatin levels in MAFLD and their association with several cardiovascular parameters. We reported no significant difference in serum adiponectin and visfatin levels between MAFLD patients and controls. Moreover, a significant directly proportional association was reported between adiponectin and E/A ratio in the univariate linear regression analysis, while the association lost significance after adjustment using multivariate regression models. Although LVEF was not significantly associated with adiponectin in univariate analysis, interestingly, a significant inversely proportional association was demonstrated after adjustment using multivariate regression models.

Several results need to be further elaborated. In our study, we evaluated hepatic steatosis using hepatic ultrasonography, known to detect hepatocytes fat deposition only when >15–20% with a sensitivity ranging between 60 and 94% and specificity between 88 and 95% [39,40], along with SteatotestTM (Biopredictive), reported to provide a non-invasive and simple quantitative estimation of liver fat deposition, with an AUROC of 0.81 (95% CI 0.79–0.83, *p* < 0.0001) [41,42]. Currently, liver biopsy remains the gold standard for identifying hepatic steatosis and quantifying liver fibrosis. Nevertheless, it is an invasive procedure with possible complications.

Similar to currently published data, serum adiponectin and visfatin levels were not significantly different between MAFLD patients and controls in our study population. A systematic review and meta-analysis assessing adiponectin levels in NAFLD concluded that, according to liver histology, serum adiponectin levels were found to be similar in NAFL patients and controls [17]. However, the authors suggested that hypoadiponectinemia may exert a significant pathophysiological role in the progression from NAFL to NASH. Another systematic review and meta-analysis evaluated serum visfatin levels in NAFLD, concluding that visfatin levels were not found to be associated with NAFLD, whether biopsy-proven or ultrasound-diagnosed, presence or severity of hepatic steatosis, liver fibrosis, lobar inflammation, or NASH [22].

Although several published studies evaluated cardiovascular parameters in NAFLD patients, these data are scarce in the current literature involving MAFLD patients [43,44]. As reported in the current literature and the newly defined MAFLD criteria, patients with MAFLD present with metabolic dysregulations and cardiovascular risk factors, including metabolic syndrome, diabetes, hypertension and dyslipidemia [9,45]. These findings were also confirmed in our results. An interesting recently published study reported differences in cardiovascular mortality and all-cause mortality in patients with NAFLD and MAFLD, where MAFLD patients presented higher cardiovascular mortality and all-cause mortality risk [13]. Nevertheless, NAFLD per se was not associated with an increased risk of all-cause deaths after metabolic risk factors adjustment. Therefore, we believe that future studies should reevaluate important markers in MAFLD patients due to possible different results between both terms, perhaps elucidating pathogenic links related to the complex cardiovascular complications associated with MAFLD.

Age, sex and BMI are important factors that should be taken into account when interpreting echocardiographic findings. Several age-related changes have been demonstrated, including alterations in the left ventricular diastolic filling without significant age-related changes in resting left ventricular systolic function, mild increase in left ventricular mass and wall thickness, a slight decrease in left ventricular internal diastolic and systolic dimensions, especially in females, significant dilation in the left atrium in both sexes, thickening in valve leaflets and atrial septum [46]. Currently, optimal adjustment of echocardiographic parameters according to body size, especially in obese patients, remains challenging [47]. As we reported in our results in MAFLD patients, several alterations in echocardiographic parameters have been found in obese patients, including LA enlargement, left ventricular hypertrophy, as well as increased cardiac output and stroke volumes representing a physiological adaptation to increased metabolic needs [47].

In our study, we found a significant association between adiponectin and E/A ratio in univariate linear regression analysis, which lost significance after multivariate analysis. Similar to our findings, Norvik et al. conducted a cross-sectional study on 1165 women and 896 men without diabetes, reporting no significant association between adiponectin and E/A ratio [48]. On the other hand, Puchałowicz et al. reported that E/A was significantly positively associated with adiponectin in coronary artery disease patients [49]. Decreased adiponectin levels in obese subjects are linked with inflammation and increased cardiovascular risk [50,51,52,53].

Moreover, although LVEF was not significantly associated with adiponectin in the univariate analysis, interestingly, we found a significant inversely proportional association after conducting the multivariate regression analysis. Similarly, several published studies reported a significant inverse correlation between adiponectin and LVEF, where adiponectin levels increase significantly as LVEF worsens [54,55]. Although adiponectin levels are not predictive of the development of heart failure in humans, several human studies demonstrated that increased circulating adiponectin levels were linked to increased mortality in patients with chronic heart failure with reduced ejection fraction (HFrEF) [56,57,58,59,60].

Visfatin was proposed to be used as a biomarker for detecting atherosclerosis, endothelial dysfunction and vascular damage [25,26]. It is also considered to present potential prognostic value. Visfatin is a crucial player in promoting atherosclerosis and vascular inflammation. Elevated serum visfatin levels were observed in acute myocardial infarction patients and were linked to the earlier onset and higher incidence of major adverse cardiovascular events (MACE) [61]. Moreover, serum visfatin levels were positively related to CAD severity in patients with high SYNTAX scores [23]. As reported in a recently published systematic review, NAFLD patients were found to have an increased acute coronary syndrome (ACS) risk, mainly in Asian subjects, with inconsistent results in North American and European populations [62].

Our study has some limitations that need to be further discussed. Causality cannot be confirmed or negated between the reported associations due to the observational study design. We were not able to perform subgroup analyses due to the enrolled modest sample size; further, adjustments using multivariate analysis might not account for some differences in basic characteristics. The increased values of hepatic steatosis and liver fibrosis scores, as well as changes in the evaluated echocardiographic parameters can partially be attributed to the increased age of MAFLD patients compared to controls. This can be due to differences in recruitment strategies, because our controls were mainly hospital staff not known to have medical illnesses. However, we partially corrected for these differences by including the MAFLD variable in our multivariate linear regression models, which is partially correlated (multicollinear) with BMI and age. Nevertheless, these adjustments cannot completely control for said differences. Furthermore, the study being observational, residual confounding can still persist. In addition, since the systolic and diastolic functions are measured by different parameters, analyzing them can increase the family-wise error rate. We cannot generalize our results as this is a single-center study conducted only on Caucasian subjects. Another limit in generalization is due to the exclusion of patients >65 years old, in order to limit confounding of comorbidities, being known that MAFLD population is relatively older. Histopathological assessment of hepatic steatosis by liver biopsy, the current gold standard, was not performed. Therefore, results should be interpreted with caution due to the above-mentioned limitations.

Our study also has several important strengths. Hepatic steatosis was assessed by combining hepatic ultrasonography and SteatoTestTM (Biopredictive), therefore improving the accuracy of predicting hepatic steatosis. Furthermore, the new criteria for MAFLD, reported to be able to identify fatty liver disease patients with increased disease progression risk, were used in our study [45]. To the best of our knowledge, this is the first study to assess serum adiponectin and visfatin levels in MAFLD patients, as well as the first to include comprehensive cardiovascular echocardiographic and Doppler ultrasound parameters and their association with adiponectin and visfatin. Due to the increasing worldwide prevalence of metabolic disorders, including fatty liver disease, as well as the associated increased CV risk, being associated with increased morbidity and mortality, we believe that the findings of our study are of clinical significance.

## 5. Conclusions

No significant association between serum adiponectin and visfatin levels was observed in MAFLD patients vs. controls. Despite the E/A ratio being significantly associated with adiponectin in the univariate analysis, this association was attenuated after performing multivariate linear regression models. Interestingly, although adiponectin levels were not associated with LVEF in the univariate analysis, a significant inversely proportional association was observed after the multivariate linear regression analysis. However, adiponectin and visfatin levels did not predict left ventricular posterior wall thickness, while visfatin levels did not predict CIMT and interventricular septal wall thickness.

In order to confirm our demonstrated results, it is necessary to conduct further observational studies involving a larger sample size on populations from different backgrounds. Hence, adiponectin can possibly play an important role in identifying incipient cardiovascular disease in MAFLD patients through the reduction and prevention of associated cardiovascular morbidity and mortality.

## Figures and Tables

**Table 1:** General characteristics of included participants.

Characteristic	Total (*n* = 80)	Control (*n* = 40)	MAFLD (*n* = 40)	*p*-Value
Age (years), median (IQR)	46 (30–57)	30 (27–42)	53.5 (48.75–59)	<0.001
Gender (male), *n* (%)	36/80 (45)	22 (55)	22 (55)	1
BMI, median (IQR)	26.4 (22.32–31.24)	22.29 (20.17–24.89)	30.78 (28.1–34.7)	<0.001
Waist circumference (cm), median (IQR)	96.5 (81.75–105.25)	82.5 (72–91.5)	104.5 (100–111)	<0.001
Metabolic syndrome, *n* (%)	33/80 (41.25)	2 (5)	31 (77.5)	<0.001
Diabetic, *n* (%)	16/80 (20)	0 (0)	16 (40)	<0.001
Impaired fasting glucose, *n* (%)	5/80 (6.25)	2 (5)	3 (7.5)	1
Hypertensive, *n* (%)	39/80 (48.75)	7 (17.5)	32 (80)	<0.001
SBP-mean (mmHg), median (IQR)	124.5 (116.38–137.25)	120.75 (112.5–126)	132.75 (122.38–147.88)	<0.001
DBP-mean (mmHg), median (IQR)	79 (74–84)	75.75 (71.25–79.12)	83 (78.38–89)	<0.001
MAP-mean (mmHg), median (IQR)	93.92 (89–101.88)	90.67 (84.42–94)	98.92 (92.79–108.62)	<0.001
Pulse pressure-mean (mmHg), median (IQR)	45.5 (41.38–52)	44.75 (40–49)	49.25 (42.38–58.5)	0.023
Pulse-mean (bpm), median (IQR)	77.5 (70.88–84.5)	79.5 (73.38–83.75)	76.75 (68–84.5)	0.366
Smoking history, *n* (%)				0.963
Smoker:	16/80 (20)	8 (20)	8 (20)	
Never smoked:	45/80 (56.25)	22 (55)	23 (57.5)	
Ex-smoker:	19/80 (23.75)	10 (25)	9 (22.5)	
LDL (mg/dL), median (IQR)	118 (90.5–158.5)	112.5 (84–140.75)	127 (99.75–166)	0.083
HDL (mg/dL), median (IQR)	48 (42.75–59.25)	54.5 (46.75–63)	44 (37.75–49.75)	<0.001
Triglycerides (mg/dL), median (IQR)	112 (79.5–154)	82.5 (69–103.5)	147.5 (115–184.5)	<0.001
Total cholesterol (mg/dL), median (IQR)	187.5 (151.75–219.25)	184 (152–215.25)	196 (146–230.25)	0.441
Fasting blood sugar (FBS) (mg/dL), median (IQR)	91 (86–100.25)	87 (82.75–91.25)	98 (89.5–123.75)	<0.001
Adiponectin (μg/mL), mean (SD)	10.92 (1.92)	11.28 (1.57)	10.56 (2.18)	0.097
Visfatin (ng/L), median (IQR)	16.91 (11.46–23.25)	14.94 (10.6–22.27)	18.18 (12.74–23.72)	0.26

IQR, interquartile range; MAFLD, metabolic-associated fatty liver disease; BMI, body mass index; DBP, diastolic blood pressure; HDL, high-density lipoprotein; LDL, low-density lipoprotein; MAP, mean arterial pressure; SBP, systolic blood pressure.

**Table 2:** Echocardiographic and Doppler ultrasound cardiovascular parameters.

Characteristic	Total (*n* = 80)	Control (*n* = 40)	MAFLD (*n* = 40)	*p*-Value
CIMT-right (mm), median (IQR)	9 (7–10)	7 (6–9)	9.5 (8–11)	<0.001
CIMT-left (mm), median (IQR)	8.5 (7–10)	7 (6–8.25)	10 (8.75–11)	<0.001
CIMT-mean (mm), median (IQR)	8.5 (7–10)	7.25 (6.5–8.62)	9.75 (8.5–11)	<0.001
Left atrial diameter (mm), median (IQR)	31 (27–34)	29 (26–31)	34 (31–36.25)	<0.001
Left ventricular diameter (mm), median (IQR)	44 (39.75–48)	42 (38.75–44.25)	45 (43–49)	0.002
Right ventricular diameter (mm), median (IQR)	23 (21–25.25)	22 (20.75–24)	25 (22–27)	0.003
LVPWT (mm), median (IQR)	10 (8–10)	8 (8–9)	10 (10–11)	<0.001
Interventricular septal wall thickness (mm), median (IQR)	9 (8–10)	8.5 (8–9)	10 (9.75–11.25)	<0.001
Interatrial septal wall thickness (mm), median (IQR)	6 (5–7)	5 (5–7)	6 (6–7)	0.018
LVEDV (mL), median (IQR)	95 (78.5–114.25)	84 (73.75–104)	103 (92–121.75)	<0.001
LVESV (mL), median (IQR)	45 (36.75–56.75)	39 (32–47)	53.5 (43.75–62.75)	<0.001
Ejection fraction (EF) (%), median (IQR)	50 (46–56.25)	52.5 (48–57.5)	48.5 (45.75–52.5)	0.011
Stroke volume (mL), median (IQR)	51 (39–57)	44 (36.75–55.95)	53 (46.75–57.25)	0.027
Cardiac output, median (IQR)	3.52 (2.88–4.32)	3.22 (2.69–4.01)	3.79 (3.05–5.13)	0.029
Early diastolic peak velocity (E (m/s)), median (IQR)	0.74 (0.62–0.86)	0.8 (0.71–0.95)	0.66 (0.58–0.78)	<0.001
Late diastolic peak velocity (A (m/s)), median (IQR)	0.51 (0.43–0.73)	0.48 (0.42–0.57)	0.71 (0.5–0.79)	<0.001
Early diastolic velocity (e′ (m/s)), median (IQR)	0.13 (0.11–0.17)	0.17 (0.14–0.2)	0.11 (0.09–0.13)	<0.001
Late diastolic velocity (a′ (m/s)), median (IQR)	0.1 (0.07–0.14)	0.1 (0.07–0.13)	0.1 (0.08–0.16)	0.265
E/A ratio, median (IQR)	1.4 (0.98–1.8)	1.72 (1.32–1.98)	1.05 (0.76–1.42)	<0.001
e′/a′ ratio, median (IQR)	1.46 (0.81–2.13)	1.67 (1.41–2.36)	0.93 (0.7–1.58)	<0.001
E/e′ ratio, median (IQR)	5.38 (4.43–6.67)	5.05 (4.04–5.62)	5.96 (4.98–7.37)	0.004

IQR, interquartile range; MAFLD, metabolic-associated fatty liver disease; CIMT, carotid intima media thickness; LVEDV, left ventricular end diastolic volume; LVEF, left ventricular ejection fraction; LVESV, left ventricular end systolic volume; LVPWT, left ventricular posterior wall thickness; SBP, systolic blood pressure.

**Table 3:** Univariate and multivariate linear regression models and multivariate quantile regression models predicting E/A ratio, LVPWT, LVEF, CIMT (mean) and interventricular septal wall thickness in relation to adiponectin or visfatin, adjusted for MAFLD, sex, diabetes, SBP, DBP, LDL and triglycerides.

Dependent Variable	Predictor	B Unadjusted	(95% CI)	*p*-Value	R2	B Adjusted	(95% CI)	*p*-Value	B-Adjusted Sandwich	(95% CI)	*p*-Value	B-Adjusted Quantile	(95% CI)	*p*-Value
E/A ratio	Adiponectin (μg/mL)	0.069	(0.0049–0.1331)	0.035	0.056	0.0455	(−0.0148–0.1059)	0.137	0.0455	(−0.0191–0.1102)	0.172	0.04	(−0.4–0.7)	0.189
LVPWT	Adiponectin (μg/mL)	−0.15	(−0.31–0.01)	0.06	0.045	−0.02	(−0.15–0.11)	0.751	−0.02	(−0.16–0.12)	0.765	−0.03	(−0.19–0.09)	0.62
	Visfatin (ng/L)	0.0002	(−0.017–0.0173)	0.984	0	0	(−0.0125–0.0125)	0.999	0	(−0.0136–0.0136)	0.999	−0.0085	(−0.0293–0.0101)	0.263
LVEF	Adiponectin (μg/mL)	−0.52	(−1.31–0.28)	0.203	0.021	−1.03	(−1.92–−0.13)	0.026	−1.03	(−1.83–−0.22)	0.015	−1.39	(−1.97–−0.60)	0.011
CIMT (mean)	Visfatin (ng/L)	−0.0031	(−0.0283–0.0221)	0.809	0.001	−0.002	(−0.0234–0.0194)	0.852	−0.002	(−0.0203–0.0163)	0.831	−0.0028	(−0.0364–0.0141)	0.870
Interventricular septal wall thickness	Visfatin (ng/L)	−0.0012	(−0.0228–0.0204)	0.909	0	−0.0024	(−0.0186–0.0138)	0.766	−0.0024	(−0.0163–0.0115)	0.732	0.0013	(−0.0216–0.0096)	0.875

CIMT, carotid intima-media thickness; DBP, diastolic blood pressure; LDL, low-density lipoprotein; LVEF, left ventricular ejection fraction; LVPWT, left ventricular posterior wall thickness; MAFLD, metabolic associated fatty liver disease; SBP, systolic blood pressure.

## Data Availability

Data supporting the reported results can be obtained by contacting A.I. or S.L.P.

## Supplementary Materials

The following are available online at https://www.mdpi.com/article/10.3390/jcm10215194/s1, Table S1: Hepatic steatosis and fibrosis evaluation of included participants. Table S2: Spearman’s correlation coefficient analyses assessing the relation between adiponectin and visfatin levels with echocardiographic and Doppler ultrasound cardiovascular parameters.
