# Supplementary material for: Relationship between Adipokines and Cardiovascular Ultrasound Parameters in Metabolic-Dysfunction-Associated Fatty Liver Disease
Source: J Clin Med. 2021 Nov 7;10(21):5194. doi: 10.3390/jcm10215194 (PMC8584895; doi:10.3390/jcm10215194)
Supplement: Supplementary file 1 [file jcm-10-05194-s001.zip › jcm-1418592-supplementary.pdf]

Table S1. Hepatic steatosis and fibrosis evaluation of included participants.

Characteristic	Total (n = 80)	Control (n = 40)	MAFLD (n = 40)	p-Value
		Hepatic Steatosis		
Hepatic steatosis (Ultrasound), n (%)	41/80 (51.25)	1 (2.5)	40 (100)	<0.001
FLI Score, median (IQR)	51.2 (11.94–79.46)	11.52 (4.42–23.88)	79.62 (65.25–87.46)	<0.001
HSI, median (IQR)	36.47 (31.49–42.07)	31.38 (29.94–33.36)	42.1 (38.38–47.08)	<0.001
		Liver Fibrosis		
APRI, median (IQR)	0.23 (0.17–0.29)	0.18 (0.16–0.26)	0.25 (0.22–0.33)	0.002
*FIB-4 Index, median (IQR) **	0.88 (0.55–1.1)	0.57 (0.42–0.92)	0.94 (0.79–1.38)	<0.001
BARD Score, median (IQR)	2 (1.75–2.25)	2 (2–2)	2 (1–3)	0.011
*NAFLD Fibrosis Score, mean (SD) **	−2.4 (1.58)	−3.23 (1.33)	−1.57 (1.37)	<0.001
		FibroMax		
*FibroTest score, median (IQR) **	0.15 (0.1–0.23)	0.12 (0.07–0.2)	0.17 (0.11–0.29)	0.018
*ActiTest score, median (IQR) **	0.08 (0.05–0.16)	0.06 (0.03–0.09)	0.11 (0.08–0.21)	<0.001
*SteatoTest score, median (IQR) **	0.4 (0.13–0.63)	0.13 (0.09–0.22)	0.64 (0.51–0.72)	<0.001
*NashTest score, median (IQR) **	0.25 (0.25–0.5)	0.25 (0.25–0.25)	0.5 (0.5–0.5)	<0.001
*AshTest score, median (IQR) **	0.01 (0–0.02)	0.01 (0–0.01)	0.02 (0.01–0.04)	<0.001

APRI – Aspartate aminotransferase-to-platelet ratio index; BARD – Body mass index, AST/ALT ratio, diabetes; FIB-4 – Fibrosis-4; FLI – Fatty liver index; HSI – Hepatic steatosis index; IQR –Interquartile range; MAFLD –Metabolic-dysfunction-associated fatty liver disease; NAFLD – Non-alcoholic fatty liver disease; * - scores that include the age in their formula, and since MAFLD group includes older subjects than controls the differences reflect this difference too.

Table S2. Spearman's correlation coefficient analyses assessing the relation between adiponectin and visfatin levels with echocardiographic and Doppler ultrasound cardiovascular parameters.

	**All subjects**		**MAFLD**		**Control**	
	**Adiponectin (μg/ml)**	**Visfatin (ng/L)**	**Adiponectin (μg/ml)**	**Visfatin (ng/L)**	**Adiponectin (μg/ml)**	**Visfatin (ng/L)**
Adiponectin (μg/ml)	1	−0.07 (0.514)	1	−0.05 (0.755)	1	−0.03 (0.876)
Visfatin (ng/L)	−0.07 (0.514)	1	−0.05 (0.755)	1	−0.03 (0.876)	1
CIMT-right (mm)	−0.03 (0.812)	−0.17 (0.13)	0.02 (0.895)	−0.3 (0.062)	0.12 (0.461)	−0.26 (0.104)
CIMT-left (mm)	0.01 (0.919)	−0.07 (0.513)	0.13 (0.433)	−0.05 (0.759)	0.19 (0.249)	−0.32 (0.042)
CIMT - mean (mm)	0.01 (0.941)	−0.12 (0.272)	0.09 (0.584)	−0.19 (0.25)	0.2 (0.211)	−0.32 (0.047)
Left atrial diameter (mm)	−0.16 (0.155)	−0.07 (0.552)	−0.22 (0.169)	0.02 (0.879)	0.04 (0.802)	−0.27 (0.097)
Left ventricular diameter (mm)	−0.18 (0.116)	−0.04 (0.694)	−0.26 (0.107)	−0.12 (0.474)	0.09 (0.573)	−0.17 (0.306)
Right ventricular diameter (mm)	−0.24 (0.029)	0.14 (0.232)	−0.29 (0.067)	0.21 (0.202)	−0.13 (0.43)	−0.09 (0.577)
LVPWT (mm)	−0.24 (0.033)	−0.09 (0.438)	−0.16 (0.311)	0.05 (0.782)	−0.08 (0.624)	−0.45 (0.003)
Interventricular septal wall thickness (mm)	−0.11 (0.317)	−0.13 (0.259)	0.07 (0.677)	−0.09 (0.566)	−0.02 (0.896)	−0.41 (0.008)
Interatrial septal wall thickness (mm)	−0.1 (0.361)	−0.01 (0.929)	−0.1 (0.551)	−0.06 (0.712)	−0.02 (0.902)	−0.01 (0.97)
LVEDV (ml)	−0.08 (0.489)	0.07 (0.512)	0 (0.979)	0.02 (0.916)	0.02 (0.898)	−0.04 (0.797)
LVESV (ml)	−0.03 (0.801)	0.07 (0.54)	−0.01 (0.97)	−0.01 (0.95)	0.11 (0.501)	0.02 (0.887)
LVEF (%)	−0.16 (0.166)	−0.04 (0.696)	−0.14 (0.384)	0.03 (0.842)	−0.33 (0.039)	−0.08 (0.63)
Stroke volume (ml)	−0.11 (0.338)	0.11 (0.348)	−0.05 (0.748)	0.12 (0.463)	−0.09 (0.571)	0.01 (0.968)
Cardiac output	−0.11 (0.319)	0.01 (0.909)	−0.05 (0.779)	−0.01 (0.972)	−0.11 (0.496)	−0.05 (0.769)
Early diastolic peak velocity - E (m/s)	0.33 (0.002)	−0.1 (0.379)	0.21 (0.186)	−0.2 (0.209)	0.45 (0.003)	0.01 (0.948)
Late diastolic peak velocity - A (m/s)	0.04 (0.713)	−0.04 (0.727)	0.34 (0.032)	−0.17 (0.283)	−0.18 (0.274)	−0.1 (0.524)
Early diastolic velocity – e′ (m/s)	0.12 (0.279)	−0.13 (0.263)	−0.08 (0.611)	−0.09 (0.568)	0.22 (0.172)	−0.07 (0.673)
Late diastolic velocity – a′ (m/s)	−0.12 (0.282)	−0.03 (0.761)	−0.08 (0.607)	0.1 (0.541)	−0.14 (0.402)	−0.28 (0.083)
E/A ratio	0.17 (0.132)	−0.04 (0.728)	−0.21 (0.196)	0.02 (0.911)	0.47 (0.002)	0.09 (0.562)
e′/a′ ratio	0.14 (0.214)	−0.02 (0.871)	0.03 (0.846)	−0.15 (0.343)	0.2 (0.218)	0.24 (0.136)
E/e′ ratio	0.12 (0.282)	0.06 (0.601)	0.18 (0.256)	−0.09 (0.592)	0.16 (0.314)	0.11 (0.495)

CIMT – carotid intima-media thickness; LVEDV – left ventricular end-diastolic volume; LVEF – left ventricular ejection fraction; LVESV – left ventricular end-systolic volume; LVPWT – left ventricular posterior wall thickness; MAFLD – metabolic associated fatty liver disease; SBP – systolic blood pressure.
